# Inhibition of Axin1 in osteoblast precursor cells leads to defects in postnatal bone growth through suppressing osteoclast formation

**DOI:** 10.1038/s41413-020-0104-5

**Published:** 2020-08-12

**Authors:** Bing Shu, Yongjian Zhao, Shitian Zhao, Haobo Pan, Rong Xie, Dan Yi, Ke Lu, Junjie Yang, Chunchun Xue, Jian Huang, Jing Wang, Dongfeng Zhao, Guozhi Xiao, Yongjun Wang, Di Chen

**Affiliations:** 1 Longhua Hospital, Shanghai University of Traditional Chinese Medicine, 725 WanPing South Road, Shanghai, 200032 China; 2 Spine Institute, Shanghai Academy of Traditional Chinese Medicine, 725 WanPing South Road, Shanghai, 200032 China; 3 Key Laboratory, Ministry of Education of China, 725 WanPing South Road, Shanghai, 200032 China; 4 Research Center for Human Tissues and Organs Degeneration, Shenzhen Institutes of Advanced Technology, Chinese Academy of Sciences, Shenzhen, 518055 China; 5 Department of Orthopedic Surgery, Rush University Medical Center, Chicago, IL 60612 USA; 6 School of Medicine, Southern University of Science and Technology, Shenzhen, 518055 China

**Keywords:** Physiology, Bone

## Abstract

Axin1 is a negative regulator of β-catenin signaling and its role in osteoblast precursor cells remains undefined. In the present studies, we determined changes in postnatal bone growth by deletion of *Axin1* in osteoblast precursor cells and analyzed bone growth in newborn and postnatal *Axin1*^*Osx*^ mice and found that hypertrophic cartilage area was largely expanded in *Axin1*^*Osx*^ KO mice. A larger number of chondrocytes and unabsorbed cartilage matrix were found in the bone marrow cavity of *Axin1*^*Osx*^ KO mice. Osteoclast formation in metaphyseal and subchondral bone areas was significantly decreased, demonstrated by decreased TRAP-positive cell numbers, associated with reduction of MMP9- and cathepsin K-positive cell numbers in *Axin1*^*Osx*^ KO mice. OPG expression and the ratio of *Opg* to *Rankl* were significantly increased in osteoblasts of *Axin1*^*Osx*^ KO mice. Osteoclast formation in primary bone marrow derived microphage (BMM) cells was significantly decreased when BMM cells were cultured with conditioned media (CM) collected from osteoblasts derived from *Axin1*^*Osx*^ mice compared with BMM cells cultured with CM derived from WT mice. Thus, the loss of Axin1 in osteoblast precursor cells caused increased OPG and the decrease in osteoclast formation, leading to delayed bone growth in postnatal *Axin1*^*Osx*^ KO mice.

## Introduction

β-catenin is a central molecule in the canonical wingless/integrated (Wnt) pathway. When Wnt ligands interact with Frizzled and low-density lipoprotein receptor-related protein 5 and 6 co-receptors, β-catenin is activated, accumulated in the cytoplasm, and translocated into the nucleus. In the nucleus, β-catenin activates transcription of downstream genes. β-catenin signaling plays important roles in bone development, postnatal bone growth, and bone remodeling. Animal studies, in which the β-catenin signaling was either inhibited or activated in chondrocytes, osteoblasts, or osteocytes, demonstrated that activation of β-catenin signaling promotes osteoblast differentiation and bone formation and inhibition of osteoclast formation and bone resorption.^1–7^ However, the detailed mechanisms of how postnatal bone growth and bone remodeling are regulated by β-catenin signaling remain unclear.

The β-catenin signaling in mesenchymal stem cells (MSCs) promoted osteoblast differentiation, inhibited chondrocyte differentiation, and enhanced endochondral ossification.^8,9^ For example, deletion of LRP6, the co-receptor of Wnts, in nestin-expressing cells caused bone mass decrease.^10^ In addition, β-catenin signaling in mature osteoblasts inhibits osteoclast formation.^11–13^ Activation of β-catenin signaling also prevented osteoblast apoptosis.^14^ In contrast, the function of β-catenin signaling in osteoblast precursor cells has not been fully investigated.

Without Wnt ligands interacting with the cell surface receptors, cytoplasmic β-catenin is degraded by the ubiquitin–proteasome system, mediated by the destruction complex in a phosphorylation-dependent manner. Axin1 and Axin2 are scaffolding proteins in the destruction complex and promote β-catenin phosphorylation and degradation. Upon Wnt ligand binding to Wnt receptors on the cell surface, the destruction complex is dissociated and β-catenin is then released from the destruction complex and subsequently translocated into the nucleus.

Several in vivo studies were conducted to determine the role of the β-catenin destruction complex in bone development.^15–17^ For example, Axin2 KO mice showed craniosynostosis and significantly increased trabecular bone mass.^18,19^ Mice lacking APC, a member in destruction complex, in osteoblasts exhibited dramatically increased bone deposition.^11^ Axin1 is also a scaffold protein in the destruction complex and is the negative regulator of β-catenin signaling.^15,17^ Axin1 was expressed ubiquitously, and the systematic deletion of *Axin1* led to early embryonic lethality in mice.^20^ Therefore, exact functions of Axin1 at different differentiation stages during MSC differentiation have not been investigated due to the limitation of the lethality of conventional deletion of *Axin1*.

To determine the potential role of Axin1 in osteoblast precursor cells at postnatal stage during bone remodeling, we generated *Axin1*^*flox*/*flox*^ mice^21^ and bred these mice with *Osx-Cre* mice to produce *Axin1*^*Osx*^ conditional KO mice. We found that loss of *Axin1* in osteoblast precursor cells mainly affects osteoclast formation in metaphyseal bone area.

## Results

### Increased expression of β-catenin in *Axin1*^*Osx*^ KO mice

**Fig. 1:**
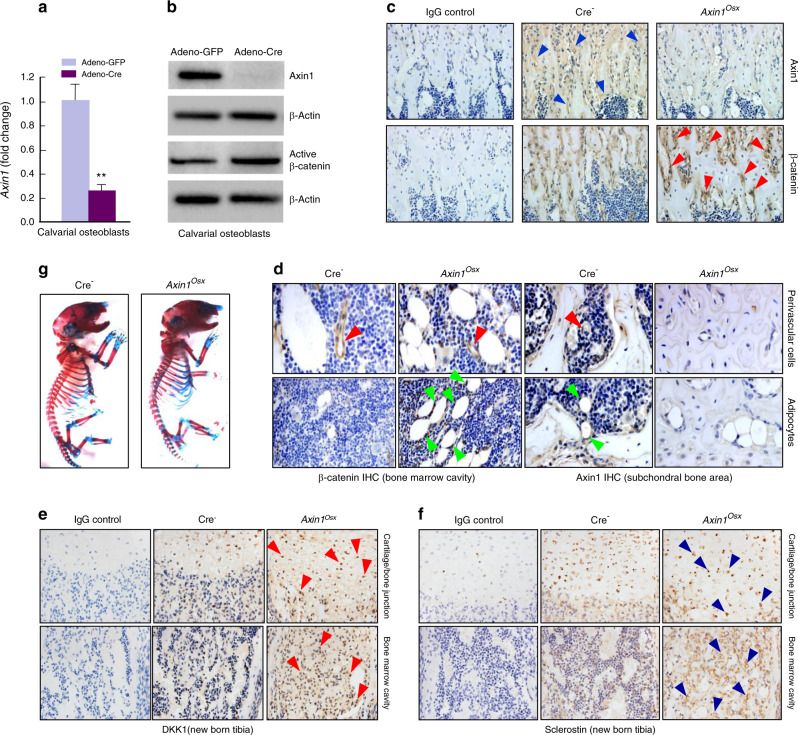
Increased β-catenin expression in *Axin1*^*Osx*^ (KO) mice. **a** *Axin1* expression was significantly decreased in *Axin1*^*flox/flox*^ calvarial osteoblasts infected with *Adeno-Cre* (*n* = 3, **P < 0.01). **b** In *Axin1*^*flox/flox*^ calvarial osteoblasts infected with *Adeno-Cre*, Axin1 protein expression was significantly decreased while active β-catenin levels were increased. **c** Results of IHC showed that Axin1 expression (blue arrowheads) was significantly decreased and β-catenin expression (red arrowheads) was significantly increased on the surface of trabecular bone of newborn *Axin1*^*Osx*^ KO mice. **d** Expression of Axin1 and β-catenin in perivascular cells (red arrowheads) and in adipocytes (green arrowheads) in bone marrow was analyzed by IHC method. **e**, **f** Expression of DKK1 (red arrowheads) and Sclerostin (blue arrowheads) in trabecular bone was analyzed by IHC method. **g** Newborn *Axin1*^*Osx*^ KO mice and Cre-negative littermates were collected and whole skeletal Alizarin red/Alcian blue staining was performed. No significant changes in skeletal structure in *Axin1*^*Osx*^ KO mice are seen

To delete *Axin1* in osteoblasts, primary osteoblasts isolated from calvariae of *Axin1*^*flox*/*flox*^ mice were infected with adenovirus-Cre recombinase. We found that *Axin1* mRNA and protein expressions were significantly decreased in calvarial osteoblasts isolated from *Axin1*^*flox*/*flox*^ mice infected with *Adeno-Cre* (Fig. 1a, b). In contrast, β-catenin expression was increased in these cells (Fig. 1b). We then bred *Axin1*^*flox/flox*^ mice with *Osx-Cre* mice and generated *Axin1*^*Osx*^ KO mice. We found that Axin1 expression was decreased while β-catenin expression was significantly increased in trabecular bone of tibiae of *Axin1*^*Osx*^ KO mice (Fig. 1c). In addition, we detected Axin1 and β-catenin expression in adipocytes and perivascular cells in the bone marrow. We found that Axin1 expression was decreased while β-catenin expression was significantly increased in adipocytes (Fig. 1d). However, we did not observe obvious changes in Axin1 and β-catenin expression in perivascular cells (Fig. 1d). We also examined changes in Wnt inhibitors by determining the expression of Dkk1 and sclerostin in trabecular bone and found that expression of both Dkk1 and sclerostin was upregulated in trabecular bone area below the growth plate in *Axin1*^*Osx*^ KO mice (Fig. 1e, f). To determine changes in skeletal structure, newborn *Axin1*^*Osx*^ KO mice and their littermate controls were collected and performed whole body Alizarin red/Alcian blue staining. We did not observe significant changes in skeletal structure in *Axin1*^*Osx*^ KO mice (Fig. 1g). In contrast, slightly delayed mineralization of calvarial bone was observed in newborn and 4-week-old *Axin1*^*Osx*^ KO mice (Fig. S1).

### Delayed endochondral bone growth in *Axin1*^*Osx*^ KO mice

**Fig. 2:**
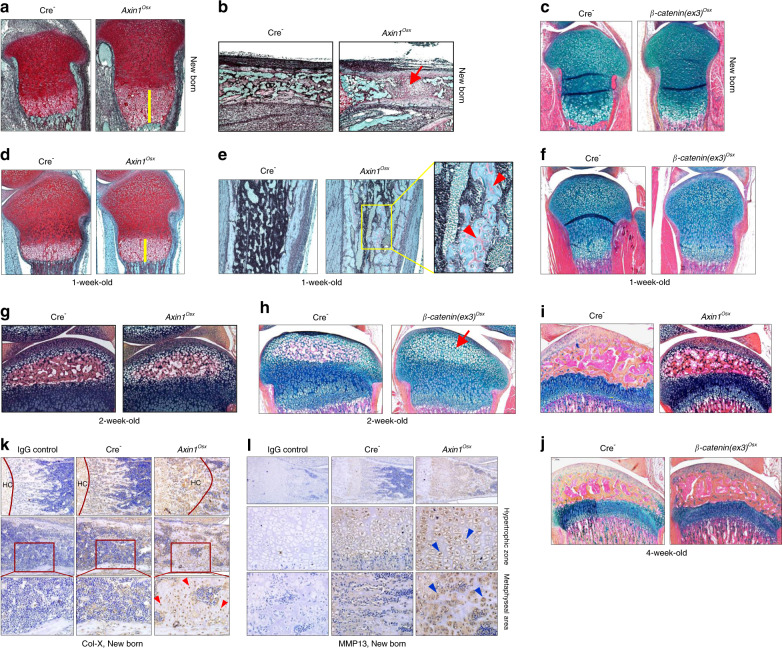
Delayed postnatal bone growth in *Axin1*^*Osx*^ KO mice. **a**, **d** Results of Safranin O/Fast green staining revealed an expanded hypertrophic zone (yellow bars) in tibial growth plates of newborn and 1-week-old *Axin1*^*Osx*^ KO mice compared with those of Cre-negative littermates. **b** Results of Alcian blue/Hematoxylin Orange G (AB/HO) staining showed that formation of a primary ossification center was delayed in newborn *Axin1*^*Osx*^ KO mice (red arrow). **e** Results of Alcian blue staining showed that trabecular bone with large amounts of uncalcified osteoid (red arrowheads) was found in 1-week-old *Axin1*^*Osx*^ KO mice. **c**, **f** No significant changes in the length and morphology of growth plate cartilage were seen in newborn and 1-week-old *β-catenin*(*ex3*)^*Osx*^ activation mice compared with their Cre-negative littermates. **g**, **h** Results of Alcian blue staining showed a slightly delayed formation of a secondary ossification center in 2-week-old *Axin1*^*Osx*^ KO mice. In contrast, a significantly delayed formation of a secondary ossification center (red arrow) was found in 2-week-old *β-catenin*(*ex3*)^*Osx*^ activation mice. **i**, **j** Results of Alcian blue staining histology showed relatively normal hypertrophic cartilage development in 4-week-old *Axin1*^*Osx*^ KO mice and in 4-week-old *β-catenin*(*ex3*)^*Osx*^ activation mice. **k**, **l** Results of IHC showed that collagen type X (Col-X)-positive hypertrophic chondrocytes (HC) (red arrowheads) and MMP13-positive hypertrophic chondrocytes (blue arrowheads) were found in the metaphyseal bone area and in the middle of the bone marrow cavity of newborn *Axin1*^*Osx*^ KO tibiae

We performed histological analyses and observed an expanded hypertrophic zone in tibial growth plates of newborn and 1-week-old *Axin1*^*Osx*^ KO mice (Fig. 2a, d). In newborn *Axin1*^*Osx*^ KO mice, the length of the hypertrophic zone is almost three times longer than that of Cre-negative mice (Fig. 2a). New bone formation in the primary ossification center was delayed in newborn *Axin1*^*Osx*^ KO mice (Fig. 2b). In 1-week-old *Axin1*^*Osx*^ KO mice, the length of the hypertrophic zone of *Axin1*^*Osx*^ KO mice was significantly longer compared with that of Cre-negative controls (Fig. 2d). Large amounts of uncalcified bone with accumulated osteoid were found in the trabecular bone area below the growth plate (Fig. 2e), suggesting defects in bone remodeling in *Axin1*^*Osx*^ KO mice. To determine if these changes are due to activation of β-catenin signaling and to compare the difference between *Axin1*^*Osx*^ KO mice and *β-catenin* conditional activation mice, we generated and analyzed newborn and 1-week-old *β-catenin*(*ex3*)^*Osx*^ activation mice. Compared with *Axin1*^*Osx*^ KO mice, we did not observe obvious expansion of hypertrophic cartilage in *β-catenin*(*ex3*)^*Osx*^ activation mice (Fig. 2c, f). These findings suggest that Axin1 may also act through a β-catenin-independent mechanism to regulate postnatal bone growth. In 2-week-old *Axin1*^*Osx*^ KO mice, we observed a slightly delayed formation of a secondary ossification center (Fig. 2g). In contrast, the formation of secondary ossification centers were significantly delayed in *β-catenin*(*ex3*)^*Osx*^ activation mice (Fig. 2h). In 4-week-old mice, it seems that growth plate cartilage development and the formation of a secondary ossification center were normal in *Axin1*^*Osx*^ KO mice (Fig. 2i) or in *β-catenin*(*ex3*)^*Osx*^ activation mice (Fig. 2j). Results of IHC analyses showed that extensively increased Col-X-positive hypertrophic chondrocytes were found in the metaphyseal bone area of newborn *Axin1*^*Osx*^ KO tibiae (Fig. 2k). Similarly, extensively increased MMP13-positive cells were also found in the expanded hypertrophic zone (Fig. 2l). This phenotype was 100% penetrated in *Axin1*^*Osx*^ KO mice.

### Impaired osteoclast formation in *Axin1*^*Osx*^ KO mice

**Fig. 3:**
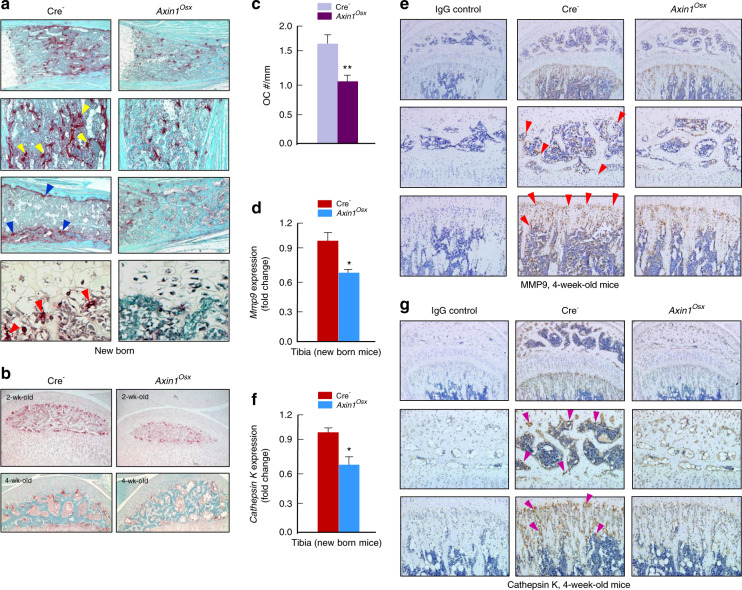
Decreased osteoclast formation in *Axin1*^*Osx*^ KO mice. **a** In newborn Cre-negative tibiae, TRAP-positive osteoclasts were found on metaphyseal bone surfaces (yellow arrowheads), the inner region of cortical bone (blue arrowheads) and the ossification front (red arrowheads). In newborn *Axin1*^*Osx*^ KO tibiae, TRAP-positive osteoclasts in the bone marrow cavity and ossification front were significantly decreased. **b** TRAP-positive osteoclasts were decreased in the subchondral bone area of 2- and 4-week-old *Axin1*^*Osx*^ KO tibiae. **c** Osteoclast numbers were quantified. The results showed significantly reduced osteoclast numbers, normalized to trabecular bone perimeters, in the subchondral bone of 4-week-old *Axin1*^*Osx*^ KO mice compared with those of Cre-negative mice (*n* = 4, **P < 0.01). **d**–**g** Real-time PCR and immunohistochemistry (IHC) data showed decreased mRNA and protein expressions of MMP9 (red arrowheads) and cathepsin K (purple arrowheads) in subchondral bone (middle panel) and metaphyseal bone (lower panel) of 4-week-old *Axin1*^*Osx*^ KO tibiae. (*n* = 4, *P < 0.05)

**Fig. 4:**
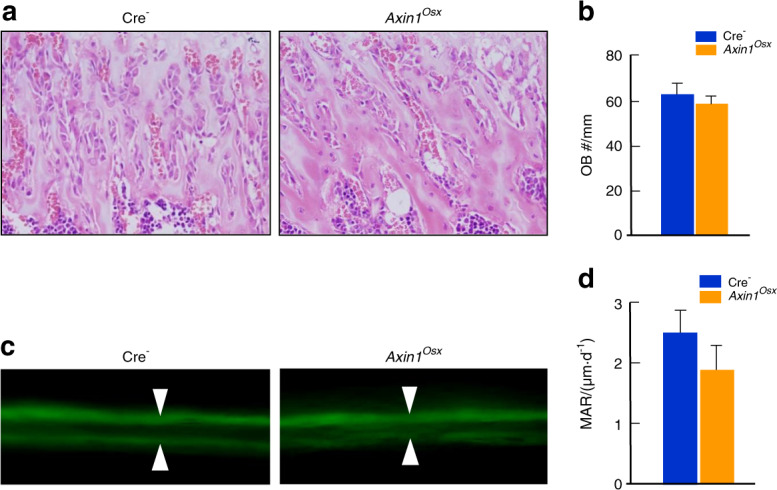
No significant change in the bone formation in *Axin1*^*Osx*^ KO mice. **a** Hematoxylin and eosin (HE) staining of trabecular bone in the metaphyseal area of 4-week-old *Axin1*^*Osx*^ KO mice and Cre-negative controls. **b** Quantification of osteoblast numbers showed no significant changes in osteoblast numbers in trabecular bone in the metaphyseal area of 4-week-old *Axin1*^*Osx*^ KO mice (*n* = 6). **c**, **d** Results of calcein labeling and the measurement of mineral appositional rates (MAR) showed cortical bone formation was slightly reduced in 4-week-old *Axin1*^*Osx*^ KO mice (*n* = 6)

Osteoclasts in growth plate can phagocytose dying hypertrophic chondrocytes and absorb the mineralized cartilage matrix. Therefore, we analyzed changes in osteoclast formation in *Axin1*^*Osx*^ KO mice. In newborn Cre-negative tibiae, large numbers of TRAP-positive osteoclasts were found on the metaphyseal bone area and the inner side of cortical bone (Fig. 3a). In addition, osteoclasts were also found in the ossification front, where osteoclasts invaded hypertrophic chondrocytes (Fig. 3a, lower left panel). In newborn *Axin1*^*Osx*^ KO tibiae, TRAP-positive osteoclasts in the bone marrow cavity and ossification front were significantly decreased (Fig. 3a, right panel). Osteoclast formation in the subchondral bone of 2- and 4-week-old *Axin1*^*Osx*^ KO tibiae was also decreased compared with that of Cre-negative tibiae (Fig. 3b). Quantification of the ratios of osteoclast numbers and trabecular bone perimeters showed reduced TRAP-positive osteoclast numbers in 4-week-old *Axin1*^*Osx*^ KO mice (Fig. 3c). Real-time PCR and IHC results also showed decreased expression of osteoclast markers, MMP9 (Fig. 3d, e) and cathpesin K (Fig. 3f, g) in both subchondral bone and metaphyseal bone of 4-week-old *Axin1*^*Osx*^ KO tibiae. By contrast, no obvious changes in osteoblast numbers were found in the metaphyseal bone of same-aged *Axin1*^*Osx*^ KO mice (Fig. 4a, b). Results of calcein labeling and quantification of mineral appositional rates (MAR) showed that bone formation of cortical bone was not significantly changed in *Axin1*^*Osx*^ KO mice (Fig. 4c, d).

### Increased *Opg* expression in *Axin1*^*Osx*^ KO mice

**Fig. 5:**
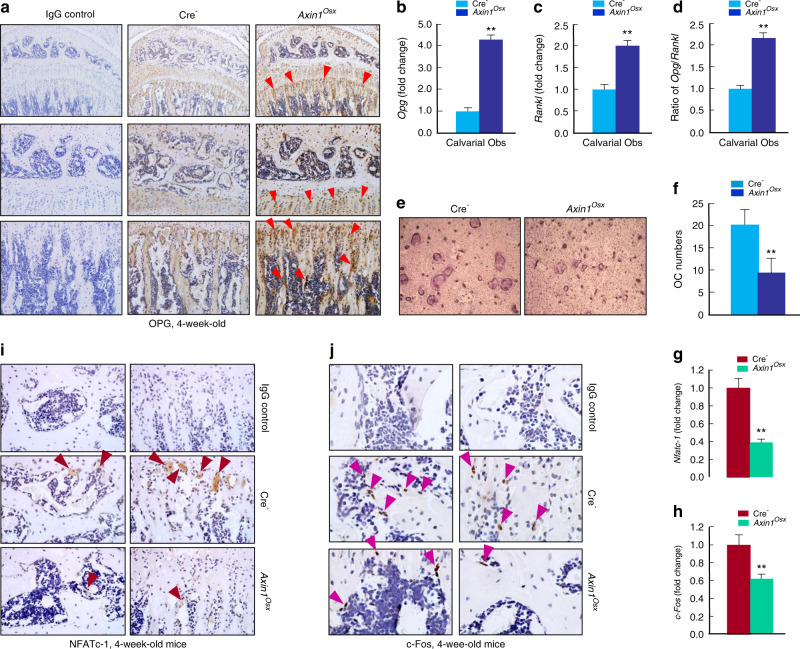
Alterations of OPG, NFATc-1, and c-Fos expression in *Axin1*^*Osx*^ KO mice. **a** Results of IHC staining showed that more osteoprotegerin (OPG)-positive cells were observed in 4-week-old *Axin1*^*Osx*^ KO mice, especially on the surface of trabecular bone (red arrowheads). **b**–**d** In calvarial osteoblasts isolated from newborn *Axin1*^*Osx*^ KO mice, both *Opg* and *Rankl* expression was increased, and the ratio of *Opg/Rankl* was significantly higher in bone marrow stromal (BMS) cells derived from *Axin1*^*Osx*^ mice than that of Cre-negative mice (*n* = 4, **P < 0.01). **e** WT BMM cells were cultured with the conditioned medium (CM) collected from cultured calvarial osteoblasts of 4-week*-*old *Axin1*^*Osx*^ KO mice and Cre-negative littermates, respectively. Osteoclast formation in the cells cultured with CM from the *Axin1*^*Osx*^ KO calvarial cells was significantly decreased compared with that in the cells cultured with CM collected from Cre-negative osteoblasts. **f** Quantification results showed decreased osteoclast numbers in the *Axin1*^*Osx*^ KO group (*n* = 6, **P < 0.01). **g**, **h** The total RNA was extracted from tibia of 4-week-old Cre-negative and *Axin1*^*Osx*^ KO mice and *Nfatc-1* and *c-Fos* mRNA expression was detected by real-time PCR (*n* = 4, **P < 0.01). **i**, **j** In tibiae of 4-week-old *Axin1*^*Osx*^ KO mice, NFATc-1-positive staining cells (brown arrowheads) and c-Fos-positive staining cells (purple arrowheads) were decreased compared with those of Cre-negative control mice

The ratio of *Opg*/*Rankl* is an important index reflecting changes in osteoclast formation. In the tibiae of Cre-negative mice, osteoprotegerin (OPG)-positive staining cells were observed on the trabecular bone surface (Fig. 5a). In *Axin1*^*Osx*^ KO mice, the number and staining intensity of OPG-positive cells were significantly increased in *Axin1*^*Osx*^ KO mice, especially on the trabecular bone surface (Fig. 5a). Osteoblasts are the important cell resource for OPG production and osteoblast precursor cells are the Osterix-expressing cells. Therefore, we examined changes in *Opg* and *Rankl* expression in primary calvarial osteoblasts of newborn *Axin1*^*Osx*^ KO mice and Cre-negative littermate controls. In calvarial osteoblasts of newborn *Axin1*^*Osx*^ KO mice, though both *Opg* and *Rankl* expressions were increased (Fig. 5b, c), the *Opg*:*Rankl* ratio was still significantly higher in *Axin1*-deficient osteoblasts than that of control osteoblasts (Fig. 5d). We then cultured wild-type (WT) bone marrow derived macrophages (BMM) cells treated with the conditioned media (CM) collected from primary osteoblasts isolated from *Axin1*^*Osx*^ KO mice and Cre-negative mice, respectively. Osteoclast formation in the BMM cells treated with CM collected from osteoblasts of *Axin1*^*Osx*^ KO mice was obviously decreased compared with the cells treated with CM collected from control osteoblasts (Fig. 5e). These findings were further confirmed by the results of osteoclast quantification (Fig. 5f). Nuclear factor of activated T-cells, cytoplasmic 1 (NFATc-1) and c-Fos are two key regulators of osteoclast differentiation upon receptor activator of nuclear factor kappa-Β ligand (RANKL) induction. Real-time PCR assay revealed that expression of *Nfatc-1* and *c-Fos* was decreased in trabecular bone of *Axin1*^*Osx*^ KO mice (Fig. 5g, h). IHC assays further demonstrated that NFATc-1- and c-Fos-expressing cells were detected in trabecular bone, and that NFATc-1 was especially highly expressed in the ossification front of the tibiae in control mice (Fig. 5i, j). The numbers of NFATc-1 and c-Fos positive cells were significantly decreased in the tibiae of *Axin1*^*Osx*^ KO mice (Fig. 5i, j).

## Discussion

Osterix is the osteoblast specific transcription factor that regulates osteoblast precursor cell differentiation and inhibits cell proliferation.^22,23^ In *Osx*-expressing osteoblast precursor cells, Wnt/β-catenin signaling activation is at a low level. *Osx-Cre* targeting cells were established by breeding *Osx-Cre* mice with *ROSA*^*mT/mG*^ reporter mice, followed by fluorescence microscopic analysis. *Osx-Cre* targeting cells were detected in trabecular bone and on the endosteal region of cortical bone; they were especially highly expressed in the area of metaphyseal bone that is close to the growth plate hypertrophic cartilage area.^22^ Consistent with these findings, when Axin1 expression was inhibited in osteoblast precursor cells using *Osx-Cre* transgenic mice, β-catenin-positive staining cells were also detected in trabecular bone and on the endosteal region of the cortical bone.

In this project we discovered that deletion of *Axin1* in osteoblast precursor cells led to β-catenin upregulation, which further resulted in increased OPG expression. Increased OPG expression could inhibit osteoclast formation, which may be responsible for the reduction of apoptosis in hypertrophic chondrocytes and decreased resorption of mineralized cartilage matrix. Wnt/β-catenin signaling controls osteoblast and osteoclast differentiation and regulates bone mass.^24,25^ In our study, osteoblast numbers and bone mass were not significantly changed in *Axin1*^*Osx*^ KO mice. In contrast, osteoclast formation was dramatically decreased when *Axin1* was deleted in *Osx*-expressing osteoblast precursor cells. In *Axin1*^*Osx*^ KO mice, β-catenin levels were increased in osteoblast precursor cells. It is known that inhibition of β-catenin signaling causes an increase in chondrocyte apoptosis during postnatal cartilage development.^26^ β-catenin, together with TCF proteins, regulates *Opg* expression in osteoblasts.^12^ Loss of *β-catenin* in mature osteoblasts resulted in a decreased ratio of OPG/RANKL, while accumulation of β-catenin signaling led to an increased ratio of OPG/RANKL.^11–13^ OPG is a decoy receptor that prevents RANKL/RANK interaction and inhibits osteoclast differentiation.^27–29^ Therefore, activation of β-catenin signaling in mature osteoblasts resulted in a decrease in osteoclast formation, while inhibition of β-catenin signaling caused an increase in osteoclast formation leading to severe osteopenia.^11–13^ In addition, β-catenin signaling in chondrocytes could also regulate osteoclast formation and bone resorption through regulation of OPG expression.^30^ In this report, we have demonstrated that deletion of *Axin1* in osteoblast precursor cells led to upregulation of β-catenin signaling, increased the ratio of OPG/RANKL and prevented osteoclast formation. We also found that *Rankl* expression in primary calvarial osteoblasts of *Axin1*^*Osx*^ mice was also upregulated. This is not consistent with previous reports that β-catenin signaling downregulated *Rankl* expression in a glucocorticoid receptor-dependent manner.^27^ This discrepancy may be due to the possibility that Axin1 may also affect other signaling molecules in addition to that of β-catenin. In fact, comparing differences in the histological results of newborn, 1-week-old and 2-week-old *Axin1*^*Osx*^ KO mice with those of *β-catenin*(*ex3*)^*Osx*^ activation mice, the phenotypes of the expanded hypertrophic zone and formation of the secondary ossification center are very different between *Axin1* KO mice and *β-catenin* activation mice, suggesting that Axin1 may also act through a β-catenin-independent mechanism to regulate postnatal bone growth.

During the endochondral bone formation process, hypertrophic chondrocytes in the growth plate undergo apoptosis and the mineralized cartilage matrix was absorbed, followed by bone formation. At the ossification front, hypertrophic chondrocytes directly contact with osteoclasts. Findings with annexin-V labeling confirmed that osteoclasts could remove dying chondrocytes by phagocytosis.^31^ Meanwhile, osteoclasts are also capable of absorbing the mineralized cartilage matrix.^32,33^ Mice lacking TRAP resulted in an expanded hypertrophic zone and disordered hypertrophic chondrocyte columns that extended into the trabecular bone region.^34^ Mice lacking RANK showed an even more severe phenotype of an expanded hypertrophic zone. The bone marrow cavity was almost filled with unabsorbed cartilage.^35,36^ In *Axin1*^*Osx*^ KO mice, we also found an expanded hypertrophic zone and unabsorbed cartilage in the bone marrow cavity, which may be caused by decreased osteoclast formation. Meanwhile, large numbers of Col-X-positive prehypertrophic and hypertrophic chondrocytes and MMP13-expressing terminally differentiated hypertrophic chondrocytes were found in the expanded hypertrophic zone of the growth plate as well as in the bone marrow cavity. This suggests that the terminal apoptosis of chondrocytes was inhibited or delayed due to the loss of *Axin1* in osteoblast precursor cells. Although Osterix was also detected in a subset of chondrocytes,^37,38^ in *Axin1*^*Osx*^ KO mice, β-catenin upregulation was found in the chondrocytes located in the upper part of the proliferative zone, but not in the hypertrophic zone. However, the phenotypes of the expanded hypertrophic zone and unabsorbed cartilage matrix observed in *Axin1*^*Osx*^ KO mice may not be primarily caused by the alteration of β-catenin signaling in hypertrophic chondrocytes. The evidence that the phenotype of the expanded hypertrophic zone was not observed in β-catenin activation mice further suggest that the phenotype observed in early postnatal *Axin1* KO mice was not caused by the activation of β-catenin signaling. The detailed mechanism of Axin1 regulation of postnatal bone growth needs to be further investigated.

In conclusion, loss of Axin1 in osteoblast precursor cells led to activation of β-catenin signaling, which in turn upregulated OPG expression and increased the ratio of OPG/RANKL. Osteoclast formation was then inhibited by OPG, which is responsible for decreased apoptosis of hypertrophic chondrocytes and reduced resorption of the mineralized cartilage matrix. However, it is not totally clear whether only a small portion of osteoblast precursor cells was affected leading to the changes in β-catenin signaling in those cells. This possibility needs to be further investigated.

## Materials and methods

### Animals

The use of animals was approved by Shanghai Laboratory Animal Use Committee. The generation of *Axin1*^*flox/flox*^ mice has been reported in previous studies.^21^ *Osx-Cre* mice were obtained from the Jackson Laboratory. *Axin1*^*Osx*^ conditional KO mice were generated by crossing *Axin1*^*flox/flox*^ mice with *Osx-Cre* transgenic mice (*Osx-Cre;Axin1*^*flox*/*flox*^). *β-catenin*(*ex3*)^*flox/-*^ mice have been used in our previous studies.^30,39^

### Isolation and culture of calvarial osteoblasts

The periosteal layers on both sides of the skull of 3-day-old mice were removed. The calvariae were transferred into a 50 mL conical tube and digested in 1 mg·mL^−1^ collagenase A (Roche, Basel, Switzerland) in serum-free αMEM in a 37 °C water bath for 15 min. After two repeated digestions in fresh collagenase A solution, the retained mixtures of collagenase A and cells were filtered into a new tube. The cell suspensions were mixed, centrifuged, and resuspended in αMEM with 10% FBS and 50 μg·mL^−1^ ascorbic acid. The suspension was finally plated in six-well culture plates at a density of 2 × 10^5^ cells per well. The media were changed every other day.

### Adenovirus mediated deletion of *Axin1* in primary calvarial osteoblasts

Primary osteoblasts were isolated from calvariae of *Axin1*^*flox*/*flox*^ mice and cultured in αMEM with 10% FBS in six-well plates. When cells reach 40% density, Adeno-Cre or Adeno-GFP (Hanbio, Shanghai, China) was added to the culture medium at a concentration of 2 × 10^8^ plaque-forming unit (PFU) per mL. The culture medium was changed 24 h after infection. The cells were collected 40 h after virus infection for real-time PCR and western blot assays.

### Real-time PCR assay

Total RNA was extracted from primary calvarial osteoblasts using a RNeasy mini kit (Qiagen, Hilden, Germany). DNAse I-treated total RNA was reverse transcripted using a RT reagent kit (Takara Bio, Tokyo, Japan). The cDNA was amplified by PCR in a total volume of 20 μL reaction solution containing 10 pmol·L^−1^ primers (primer names and sequences are listed in Table 1).

**Table 1:** Names and sequences of PCR primers

Gene Name	Sequence
*β-actin* (forward)	5′-GGAGATTACTGCCCTGGCTCCTA-3′
*β-actin* (reverse)	5′-GACTCATCGTACTCTGCTTGCTG-3′
*Axin1* (forward)	5′-GGACCTCGGAGCAAGTTTCA-3′
*Axin1* (reverse)	5′-GGTTGACAGGCCTCGAATCA-3′
*Opg* (forward)	5′-CAGAGCGAAACAC AGTTTG-3′
*Opg* (reverse)	5′-CACACAGGGTGACATCTATTC-3′
*Rankl* (forward)	5′-CAGGTTTGCAGGACTCGAC-3′
*Rankl* (reverse)	5′-AGCAGGGAAGGGTTGGACA-3′
*Mmp9* (forward)	5′-CCATGCACTGGGCTTAGATCA-3′
*Mmp9* (reverse)	5′-GGCCTTGGGTCAGGCTTAGA-3′
*Cathepsin K* (forward)	5′-CAGCAGAACGGAGGCATTGA-3′
*Cathepsin K* (reverse)	5′-CTTTGCCGTGGCGTTATACATACA-3′
*NFATc-1* (forward)	5′-CCGTTGCTTCCAGAAAATAACA-3′
*NFATc-1* (reverse)	5′-TGTGGGATGTGAACTCGGAA-3′
*c-Fos* (forward)	5′-CGGGTTTCAACGCCGACTAC-3′
*c-Fos* (reverse)	5′-AAAGTTGGCACTAGAGACGGACAGA-3′

### Whole skeleton Alizarin red and Alcian blue staining

After removing skin and adipose tissues, the skeletons were fixed in 95% ethanol for 2 days followed by fixation in acetone for another day. The skeletons were then stained with 0.015% Alcian blue and 0.005% Alizarin red for 3 days. Pictures were taken after most of the soft tissue was digested in 1% potassium chloride solution.

### Histological analysis

Tibial samples were fixed in 4% paraformaldehyde, decalcified, dehydrated, and embedded in paraffin. 4-μm thick serial midsagittal sections of tibias were cut and stained with Alcian blue/HEG (ABH) and Safranin O/Fast green (SOF). Histomorphometric analysis was performed using an Olympus BX50 microscope (Olympus, Tokyo, Japan) and Image-Pro Express software (Media Cybernetics, Rockville, MD, USA). TRAP-positive osteoclast numbers were quantified.

### Calcein labeling assay

1 mg·mL^−1^ calcein in saline was injected into 3-week-old mice (i.p. injection, 10 mg·kg^−1^) followed by a second injection 4 days later. Tibiae of 4-week-old mice were fixed in 4% paraformaldehyde and dehydrated in a series of ethanol (75%–100%). Samples were embedded in methyl methacrylate and 100-μm thick serial midsagittal sections were cut. The fluorescence signal of the cortical bone was observed using an Olympus BX50 microscope (Olympus). The distances of two calcein labels were measured with DP Manager software (Olympus) to determine the MAR.

### TRAP staining

Paraffin sections of newborn, 2- and 4-week-old tibias were rehydrated and incubated with a 0.4 mg·mL^−1^ Napthol-Ether solution/basic stock incubation solution at 37 °C for 30 min. A 0.04 g·mL^−1^ sodium nitrite solution and a 0.05 g·mL^−1^ pararosaniline dye/2 N hydrochloric acid solution was mixed and added to the basic stock incubation solution and the paraffin sections were incubated in this mixture for 10 min.

### Immunohistochemistry (IHC) staining

Paraffin sections of 4-week-old tibiae were rehydrated and digested in 0.1% trypsin for 10 min at the room temperature, and then treated with 3% H_2_O_2_ for 20 min. Sections were incubated with primary antibodies in PBS overnight at 4 °C. Col-X, MMP13, MMP9, cathepsin K, OPG, DKK1, and sclerostin antibodies were obtained from Abcam (Cambridge, MA, USA). Axin1 and β-catenin antibodies were obtained from Sigma (St. Louis, MO, USA). NFATc-1 and c-Fos antibodies were obtained from Santa Cruz Biotechnology (Santa Cruz, CA, USA). Negative control sections were incubated with IgG (Beyotime Biotechnology, Shanghai, China). A Polink-2 plus polymer HRP detection kit (PV-9001, ZSGB-BIO, Shanghai, China) was used for incubation with secondary antibody and horseradish peroxidase (HRP)-streptavidin.

### In vitro osteoclast differentiation assay

BMM cells were isolated from long bone of 1-month-old WT mice and plated into 24-well culture plates at a density of 4 × 10^5^ cells per well and cultured in αMEM with 10% FBS. Cells were treated with 20 ng·mL^−1^ macrophage colony-stimulating factor (M-CSF) for 3 days, and then switched to the medium with 10 ng·mL^−1^ M-CSF and 50 ng·mL^−1^ RANKL for 7 days. To test *Axin1*^*Osx*^ CM, BMM cells were treated with CM collected from cultured calvarial osteoblasts isolated from *Axin1*^*Osx*^ KO mice and Cre-negative controls for 7 days. The culture medium was changed every 3 days. TRAP staining was performed using a TRAP assay kit (Sigma, St. Louis, MO, USA).

### Statistical analysis

An unpaired Student’s *t* test was performed for experiments involving two groups. *P* < 0.05 was considered statistically significant.

## Supplementary information

Supplementary Figure S1

Supplementary figure legend

## Data Availability

All data and materials used in the analysis are available to any researcher for purposes of reproducing or extending the analysis.
