# Supplementary figures and images for: Inhibition of Axin1 in osteoblast precursor cells leads to defects in postnatal bone growth through suppressing osteoclast formation
Source: Bone Res. 2020 Aug 12;8:31. doi: 10.1038/s41413-020-0104-5 (PMC7424530; doi:10.1038/s41413-020-0104-5)

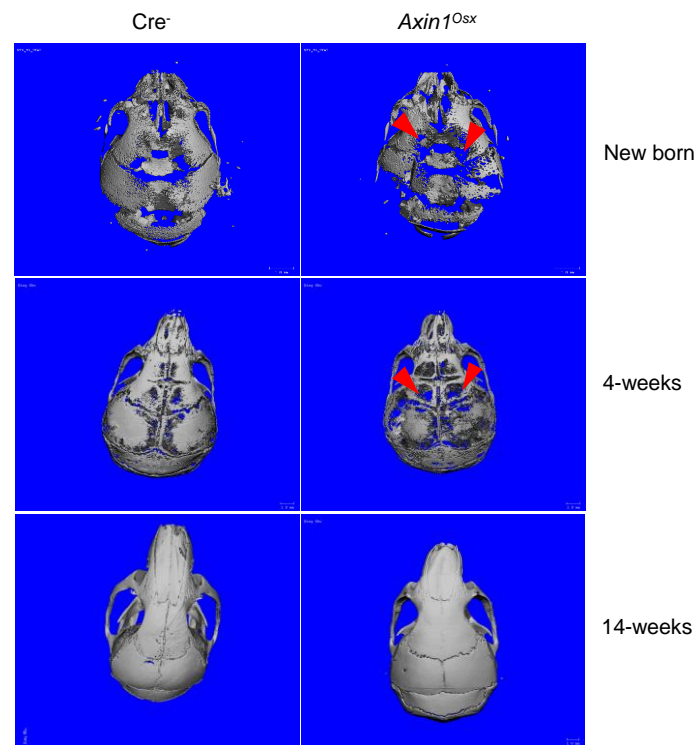

Figure S1

Supplement: Supplementary file 1 — Supplementary Figure S1 [file 41413_2020_104_MOESM1_ESM.pdf]
