# Supplementary material for: Inhibition of Axin1 in osteoblast precursor cells leads to defects in postnatal bone growth through suppressing osteoclast formation
Source: Bone Res. 2020 Aug 12;8:31. doi: 10.1038/s41413-020-0104-5 (PMC7424530; doi:10.1038/s41413-020-0104-5)
Supplement: Supplementary file 2 — Supplementary figure legend [file 41413_2020_104_MOESM2_ESM.docx]

**Figure S1.** **The mineralization of calvarial bone was delayed in *Axin1^Osx^* KO mice**. μCT analysis showed that the mineralization of calvarial bone was delayed in new born and 4-week-old *Axin1^Osx^* knockout (KO) mice.
